# The relative importance of frailty, physical and cardiovascular function as exercise-modifiable predictors of falls in haemodialysis patients: a prospective cohort study

**DOI:** 10.1186/s12882-020-01759-z

**Published:** 2020-03-14

**Authors:** Tobia Zanotto, Thomas H. Mercer, Marietta L. van der Linden, Robert Rush, Jamie P. Traynor, Colin J. Petrie, Arthur Doyle, Karen Chalmers, Nicola Allan, Ilona Shilliday, Pelagia Koufaki

**Affiliations:** 1 Centre of Health, Activity and Rehabilitation Research, School of Health Sciences, Queen Margaret University, Edinburgh, EH21 6UU UK; 2 Renal and Transplant Unit, Queen Elizabeth University Hospital, Glasgow, UK; 3 Department of Cardiology, Monklands Hospital, Airdrie, UK; 4 Renal Unit, Victoria Hospital, Kirkcaldy, UK; 5 Renal Unit, Monklands Hospital, Airdrie, UK

**Keywords:** Stage 5 chronic kidney disease, Haemodialysis, Frailty, Physical function, Baroreflex function, Blood pressure, Falls

## Abstract

**Background:**

Stage 5 chronic kidney disease (CKD-5) patients on haemodialysis (HD) are at high risk of accidental falls. Previous research has shown that frailty is one of the primary contributors to the increased risk of falling in this clinical population. However, HD patients often present with abnormalities of cardiovascular function such as baroreflex impairment and orthostatic dysregulation of blood pressure (BP) which may also be implicated in the aetiology of falling. Therefore, we aimed to explore the relative importance of frailty and cardiovascular function as potential exercise-modifiable predictors of falls in these patients.

**Methods:**

Ninety-three prevalent CKD-5 patients on HD from three Renal Units were recruited for this prospective cohort study, which was conducted between October 2015 and August 2018. At baseline, frailty status was assessed using the Fried’s frailty phenotype, while physical function was evaluated through timed up and go (TUG), five repetitions chair sit-to-stand (CSTS-5), objectively measured physical activity, and maximal voluntary isometric strength. Baroreflex and haemodynamic function at rest and in response to a 60° head-up tilt test (HUT-60°) were also assessed by means of the Task Force Monitor. The number of falls experienced was recorded once a month during 12 months of follow-up.

**Results:**

In univariate negative binomial regression analysis, frailty (RR: 4.10, 95%CI: 1.60–10.51, *p* = 0.003) and other physical function determinants were associated with a higher number of falls. In multivariate analysis however, only worse baroreflex function (RR: 0.96, 95%CI: 0.94–0.99, *p* = 0.004), and orthostatic decrements of BP to HUT-60° (RR: 0.93, 95%CI: 0.87–0.99, *p* = 0.033) remained significantly associated with a greater number of falls. Eighty falls were recorded during the study period and the majority of them (41.3%) were precipitated by dizziness symptoms, as reported by participants.

**Conclusions:**

This prospective study indicates that cardiovascular mechanisms implicated in the short-term regulation of BP showed a greater relative importance than frailty in predicting falls in CKD-5 patients on HD. A high number of falls appeared to be mediated by a degree of cardiovascular dysregulation, as evidenced by the predominance of self-reported dizziness symptoms.

**Trial registration:**

ClinicalTrials.gov (trial registration ID: NCT02392299; date of registration: March 18, 2015).

## Background

Stage 5 chronic kidney disease (CKD-5) patients receiving maintenance haemodialysis (HD) are at high risk of falls and fall-related injuries [1–3], with incidence rates being 1.5 to 3.5 times higher than community-dwelling older adults [4–6]. The clinical implications of an accidental fall can be devastating in this patient population who are already multi-comorbid. Several prospective cohort studies conducted in HD patients have concluded that those who fell had a 2.1 to 3.5 times higher risk of admission to nursing homes, hospitalisation, and death [7, 8]. Previous research has shown that factors such as older age, comorbidity, polypharmacy and frailty seem to be principal factors implicated in the aetiology of falls in these patients [1, 2, 4, 7].

The prevalence of physical frailty in the CKD-5 population is 36.8% and is approximately five-fold higher than in community-dwelling older adults [9, 10]. From a preventive and rehabilitative point of view, this syndrome is a potentially modifiable risk factor, as previous studies conducted in HD populations have shown that single frailty components, such as low physical activity, muscle strength, and slow gait speed can be improved following exercise-based interventions [11]. Although frailty has been consistently shown to be associated with falls in HD patients [4, 12, 13], few studies have explored the relationship between objective frailty-related physical function measures and falls [1, 2, 4, 12, 14, 15]. Results from these studies seem to indicate that, in predicting falls, composite indices of physical function [14, 15] may be more useful than single component assessments [2]. However, the identification of which individual frailty/physical function components may be more closely associated with falls is of paramount importance, as this would eventually allow exercise-based rehabilitation programs to be tailored to reduce the occurrence of falls in CKD-5 patients on HD. Along with frailty, cardiovascular disease (CVD) is also highly prevalent in CKD-5 patients and impaired cardiovascular function is associated with multiple adverse clinical outcomes [16]. A few studies have also begun to better explore the link between clinical outcomes such as falls and cardiovascular dysregulation in this patient group [5, 17, 18]. In particular, it appears that the CKD-5 population on dialysis may be at risk of hypotension that can lead to postural dizziness, and potentially falls. This is further exacerbated by the combined effect of autonomic failure and the significant fluid shifts associated with dialysis [17]. Two studies reported that low pre-dialysis blood pressure (BP) was associated with increased odds of falling [2, 19], and a recent investigation by Kono et al. [15] highlighted a significant relationship between intra-dialytic hypotension and falls in HD patients. In addition, we recently postulated that a drop in BP in response to a passive orthostatic challenge, as well as impaired baroreflex function may be linked to an increased risk of falling in HD patients [18]. All of these observations seem to indirectly suggest that impaired BP control, to maintain haemodynamic stability, might be implicated as an additional factor in the aetiology of falls in the CKD-5 population on dialysis, due to the CKD-related autonomic failure and/or the dialysis-related hypotension. From a rehabilitation/therapeutic perspective, this could have important translational impact, since the short-term regulation of BP, as assessed by means of baroreflex function, has been shown to improve following active interventions such as exercise [20].

The objective of this investigation was to explore the relative importance of frailty and cardiovascular function as potential exercise-modifiable predictors of falls in CKD-5 patients on HD. We hypothesised that 1) frailty and cardiovascular function would be associated with a higher number of falls, and that 2) modelling the risk of falling by adding a cardiovascular function variable to frailty would improve the prediction of falls sustained and recorded over a 12-month period in CKD-5 patients on HD.

## Methods

### Study design

An observational prospective study design was used to investigate the association between potential predictors of falls (e.g. frailty, cardiovascular function) and the number of falls sustained over a 12-month follow-up period in CKD-5 patients on HD.

### Study setting

The study was conducted in three National Health Service (NHS) Renal Units located in North Lanarkshire and Fife, United Kingdom, between October 2015 and August 2018. Recruitment started in October 2015 and continued on a rolling basis until December 2017. All potential predictors and clinical variables were assessed (during a single session of 2 hours duration) at baseline for each participant between October 2015 and December 2017, while the follow-up period ran from November 2015 to August 2018.

This research project abided by the ethical principles for medical research involving human participants, as set out by the world medical association declaration of Helsinki, and received ethical approval by the West of Scotland NHS and Queen Margaret University Research Ethics Committees (NHS REC reference number: 15/WS/0079). This research project was prospectively registered in ClinicalTrials.gov (trial registration ID: NCT02392299; date of registration: March 18, 2015).

### Study participants

Prevalent ambulatory CKD-5 patients stable on HD therapy for at least 3 months, fluent in spoken and written English, and ≥ 18 years of age were considered eligible to participate in the study.

Exclusion criteria were lower limb amputation without prosthesis, unstable dialysis and medication treatment, unstable cardiac condition, clinically severe left ventricular outflow obstruction, suspected or known aneurysm, critical mitral stenosis, critical cerebrovascular stenosis, critical proximal coronary artery stenosis, pregnancy and severe cognitive impairment.

All patients who agreed to take part in the study provided written informed consent.

### Measurements

#### Demographic and clinical characteristics

Patient demographics (age, gender, height, weight, body mass index), and clinical characteristics (dialysis vintage, Charlson comorbidity index, medications) were extracted from the patients’ medical records. Height and weight were measured on the assessment day.

#### Falls

A fall was operationally defined as an unexpected event in which the participant comes to rest on the ground, floor, or lower level [21]. One researcher (TZ) and two research nurses (KC and NA) administered a customised falls questionnaire to all study participants during dialysis, once a month following baseline assessment, for a period of 12 months. Information recorded included number of falls experienced (if any), circumstances surrounding these falls (i.e. location, activities undertaken at the time of fall, precipitating factors), and their consequences (i.e. hospitalisation, injuries, emergency services etc.). A patient was classified as a “faller” if he/she reported at least one fall during the 12-month longitudinal follow-up.

#### Frailty

A modified version of the Fried’s frailty phenotype was used to assess frailty [10]. Patients were classified as frail if they met at least three of the following conditions: 1) low self-reported levels of physical activity (PA), assessed by means of the short-form international physical activity questionnaire (IPAQ-SF) [22] (total Kcal/week below an established threshold [10]); 2) low gait speed, assessed as walking time of 15 ft above an established threshold [10]; 3) low strength, assessed by means of an isometric handgrip test below an established threshold [10]; 4) self-reported exhaustion, assessed by means of the SF-36 questionnaire (vitality score <55) [23]; 5) unintentional weight loss ≥10 lbs. in the previous 12 months (ascertained through medical records).

#### Additional objective physical function and physical activity measures

1) The 3-m timed up and go (TUG) test was used to assess dynamic balance [24]. 2) The 5 repetitions chair sit to stand test (CSTS-5) was used to assess lower extremity muscle power [25]. 3) Maximal voluntary isometric handgrip and leg extension strength were assessed using a hydraulic hand dynamometer (Jamar Patterson Medical Ltd., USA) and a digital myometer (MIE, Medical Research Ltd., Leeds, UK): participants performed three trials interspersed by a one-minute rest and the average of these was taken for analysis purposes [26–28]. 4) Objective measures of PA patterns were obtained using the activPal monitor (PAL Technologies Ltd., Glasgow, UK). Participants wore this device on the anterior aspect of the thigh for seven consecutive days and the average daily time spent standing, number of daily steps, and number of daily sit to stands were recorded and summarised using the manufacturer’s proprietary software [29].

#### Cardiovascular function

The arterial baroreflex was assessed during quiet rest (in supine position) and in response to a five-minute head-up tilt test at 60° (HUT-60°) [18]. The Task Force Monitor 3040i (CNSystems, Graz, Austria), was used for the non-invasive measurement of all haemodynamic and baroreflex variables [30–32]. Stroke volume (SV), cardiac output (CO), and total peripheral resistance (TPR) were recorded by means of impedance cardiography (ICG). Heart rate (HR), R-R interval (RRI), continuous systolic (contSBP) and diastolic (contDBP) BP were measured by means of 6-lead electrocardiography (ECG) and continuous photoelectric plethysmography.

Baroreflex function variables taken for the analysis were i) number of up (up-events) and down (down-events) baroreceptor events, defined as the simultaneous coupling of a contSBP ramp (+/− 1 mmHg for 3 consecutive beats) with either an increase or decrease of the RRI of at least 4 ms. The total number of baroreceptor events (total-events) were then calculated as the sum of all down-events and up-events, ii) the down/up/total baroreceptor effectiveness index (BEI), representing the ratio of occurred down/up/total baroreceptor events and detected down/up/total BP ramps expressed as a percentage [33], and iii) baroreflex sensitivity (BRS), the average slope of the regression lines between the RRIs and the contSBP values resulting from every baroreceptor event [34]. All baroreflex and haemodynamic assessments were performed on a non-dialysis day, during the midweek interval, in order to minimise the influence of fluid and electrolyte shifts on data collected.

### Statistical analysis

Statistical analyses were performed with SPSS (Version 23.0 for Windows, SPSS Inc., Chicago, IL). The Kolmogorov-Smirnov Test (K-S) was used to assess whether data were normally distributed. Differences between fallers and non-fallers in demographic, clinical, frailty, and cardiovascular characteristics were analysed by means of a Chi-Squared test for categorical variables, and by either Mann-Whitney U or independent t-tests, as appropriate, for continuous variables.

The association between baseline factors and the number of falls recorded during follow-up was analysed by means of negative binomial regression with an overdispersion parameter estimated on the actual distribution of the dependent variable (i.e. number of falls). All frailty and cardiovascular factors were initially entered in a univariate negative binomial regression model, and those factors reaching statistical significance (*p*-value ≤ .05) were taken forward to the multivariate stage. The multivariate analysis consisted of a first model (Model 1), in which all factors were adjusted for clinical confounders, while a second model was designed to assess the second research hypothesis. In this second model (Model 2), frailty and physical function factors were adjusted for all variables in Model 1 and for the cardiovascular function variable showing the highest correlation (R^2^) with number of falls, while cardiovascular factors were adjusted for all variables in Model 1 and for frailty. In a further analysis, we calculated the relative change in Akaike’s Information criterion (AIC) to explore the goodness of fit of negative binomial regression models where only frailty and cardiovascular function variables were entered simultaneously.

Sensitivity analyses were undertaken using the receiver operating characteristic (ROC) analysis of those potential factors associated with falls (yes or no), and the area under the curve (AUC) was used to compare the predictive ability of frailty alone with a model composed of frailty and a cardiovascular predictor. Statistical limits for significance were set at an alpha level of *p* ≤ .05.

## Results

### Study participants

Ninety-three patients undergoing outpatient HD therapy at the Renal Units provided written informed consent. Seventeen patients (18.3%) did not complete baseline assessments due to i) change of mind or inability to schedule an assessment appointment within a couple of months from consent (*n* = 7; 7.5%), ii) deterioration of health conditions (*n* = 6; 6.5%) and iii) death (*n* = 4; 4.3%). The remaining 76 patients completed all baseline assessments and were enrolled in the study. Seven participants (9.2%) were lost to follow-up due to renal transplantation (*n* = 4; 5.3%) and death (*n* = 3; 3.9%). Therefore, 69 patients were entered in the final analysis.

### Demographic and clinical characteristics

The demographic and clinical characteristics of study participants are summarised in Table 1. Fallers were more likely to be prescribed antidepressants, and had significantly higher CRP compared to non-fallers. No statistically significant differences in other clinical characteristics were detected.

**Table 1:** Demographic and clinical characteristics of study participants: results are expressed as percentages for categorical variables and mean ± standard deviation for continuous data

Variables	All patients (69)	Fallers (26)	Non-fallers (43)	***P***-value
**Sociodemographic characteristics**				
Sex (% M)	55.1	42.3	62.8	0.097
Age (years)	61.7 ± 13.3	58.3 ± 14.1	63.8 ± 12.6	0.151
Weight (kg)	80.3 ± 18.7	75.7 ± 18.6	83.1 ± 18.5	0.114
Height (cm)	166.2 ± 9	166 ± 9	166.3 ± 9	0.892
BMI (kg * m^−2^)	29.1 ± 6.4	27.6 ± 7	30 ± 5.9	0.144
**Clinical history**				
Dialysis vintage (days)	713 ± 714	732 ± 636	701 ± 765	0.465
CCI (score)	5.3 ± 2.2	5.1 ± 2.1	5.4 ± 2.3	0.639
Primary renal disease (%)				
*Diabetic nephropathy*	25	26.9	23.8	0.773
*Glomerulonephritis*	19.1	19.2	19	1.000
*Polycystic kidney*	8.8	0	14.3	0.075
*Renovascular or hypertensive*	8.8	0	14.3	0.075
*Other*	20.6	23.1	19	0.690
*Uncertain aetiology*	17.6	30.8	9.5	0.046
Type of vascular access (%)				
*Arteriovenous fistula*	64.7	61.5	66.7	0.667
*Central-venous*	35.3	38.5	33.3	0.667
Inter-dialytic weight gain (kg)	1.6 ± 1.2	1.6 ± 1	1.6 ± 1.4	0.923
Ultrafiltration rate (mL/kg/h)	6.5 ± 2.4	7.1 ± 2.5	6.1 ± 2.3	0.095
HD treatment time (h)	4.1 ± 0.5	4.1 ± 0.4	4.2 ± 0.5	0.395
**Prescribed medications**				
Medications (n°)	11.8 ± 3.4	12.4 ± 3.3	11.5 ± 3.5	0.262
Beta blockers use (%)	50	42.3	54.8	0.318
ACE-inhibitors use (%)	7.4	7.7	7.1	1.000
Ca-channel blockers use (%)	57.4	50	61.9	0.335
AngII-receptor antagonists use (%)	16.2	11.5	19	0.512
Alpha blockers use (%)	32.4	38.5	28.6	0.397
Antihypertensive use (%)	83.8	80.8	85.7	0.737
> 1 antihypertensive use (%)	51.5	42.3	57.1	0.234
Opiates use (%)	19.1	11.5	23.8	0.342
Antidepressants use (%)	33.8	50	23.8	0.027
Diuretics use (%)	33.8	23.1	40.5	0.141
**Laboratory values**				
Hb (g/dL)	11.2 ± 1.2	11 ± 1	11.2 ± 1.2	0.317
CRP (mg/L)	25.8 ± 45.4	37.6 ± 59.2	18.3 ± 32.7	0.027
Bicarbonate (mmol/L)	21.2 ± 3.2	21.5 ± 3.1	21.1 ± 3.4	0.546
Na (mmol/L)	139 ± 2.8	138.4 ± 3.3	139.4 ± 2.4	0.388
K (mmol/L)	4.6 ± 0.7	4.6 ± 0.7	4.6 ± 0.6	0.992
Urea (mg/dL)	16.4 ± 5.1	15.7 ± 6.1	16.7 ± 4.5	0.276
Phosphate (mmol/L)	1.5 ± 0.6	1.5 ± 0.6	1.5 ± 0.5	0.978
PTH (ρmol/L)	27.2 ± 32.1	28.9 ± 43.4	26.2 ± 23.1	0.586
Albumin (g/L)	37.1 ± 4.3	36.1 ± 5.2	37.7 ± 3.7	0.146
Adjusted calcium (mmol/L)	2.4 ± 0.1	2.3 ± 0.1	2.4 ± 0.1	0.511
URR (%)	71.1 ± 6.2	71.1 ± 7.7	71 ± 5.1	0.994
Creatinine (μmol/L)	638.1 ± 161.3	592 ± 175.7	666 ± 147.1	0.064

Abbreviations: *BMI* body mass index, *CCI* Charlson comorbidity index, *HD* haemodialysis, *ACE* angiotensin-converting enzyme, *Ca* calcium, *AngII* angiotensin II, *Hb* hemoglobin, *CRP* C-reactive protein, *Na* sodium, *K* potassium, *PTH* parathyroid hormone, *URR* urea reduction ratio

### Falls

**Fig. 1:**
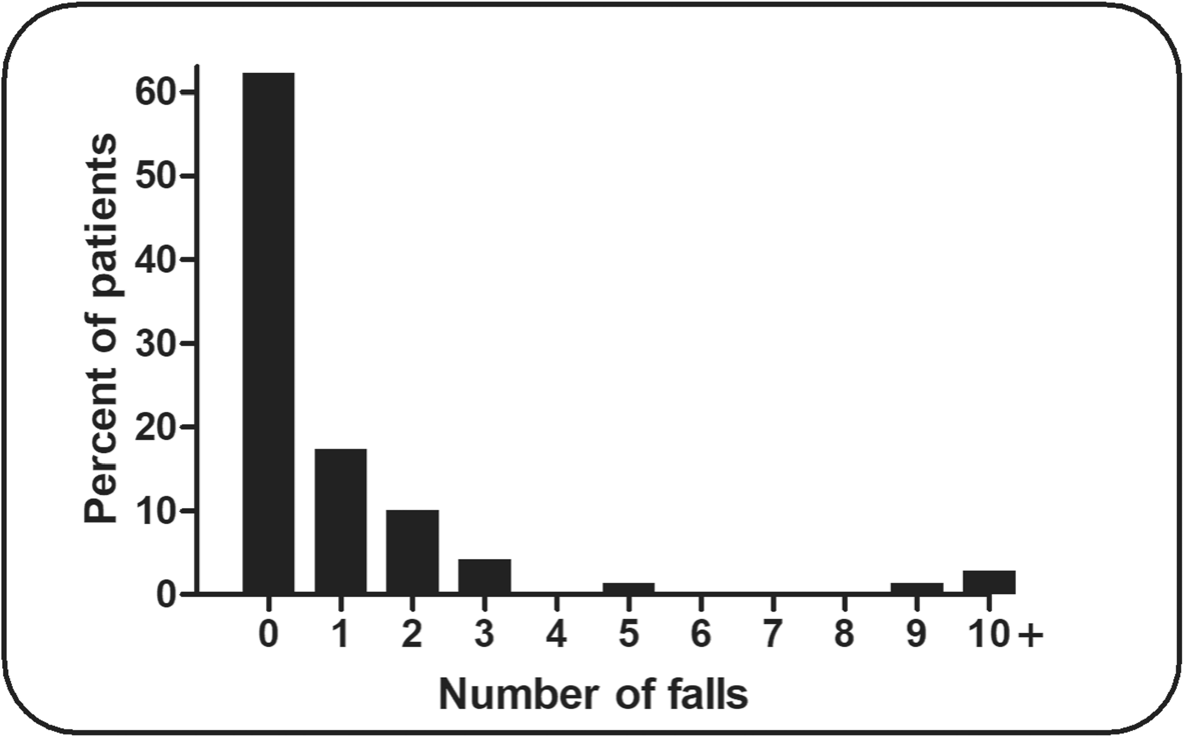
Distribution of number of falls in the study participants

**Fig. 2:**
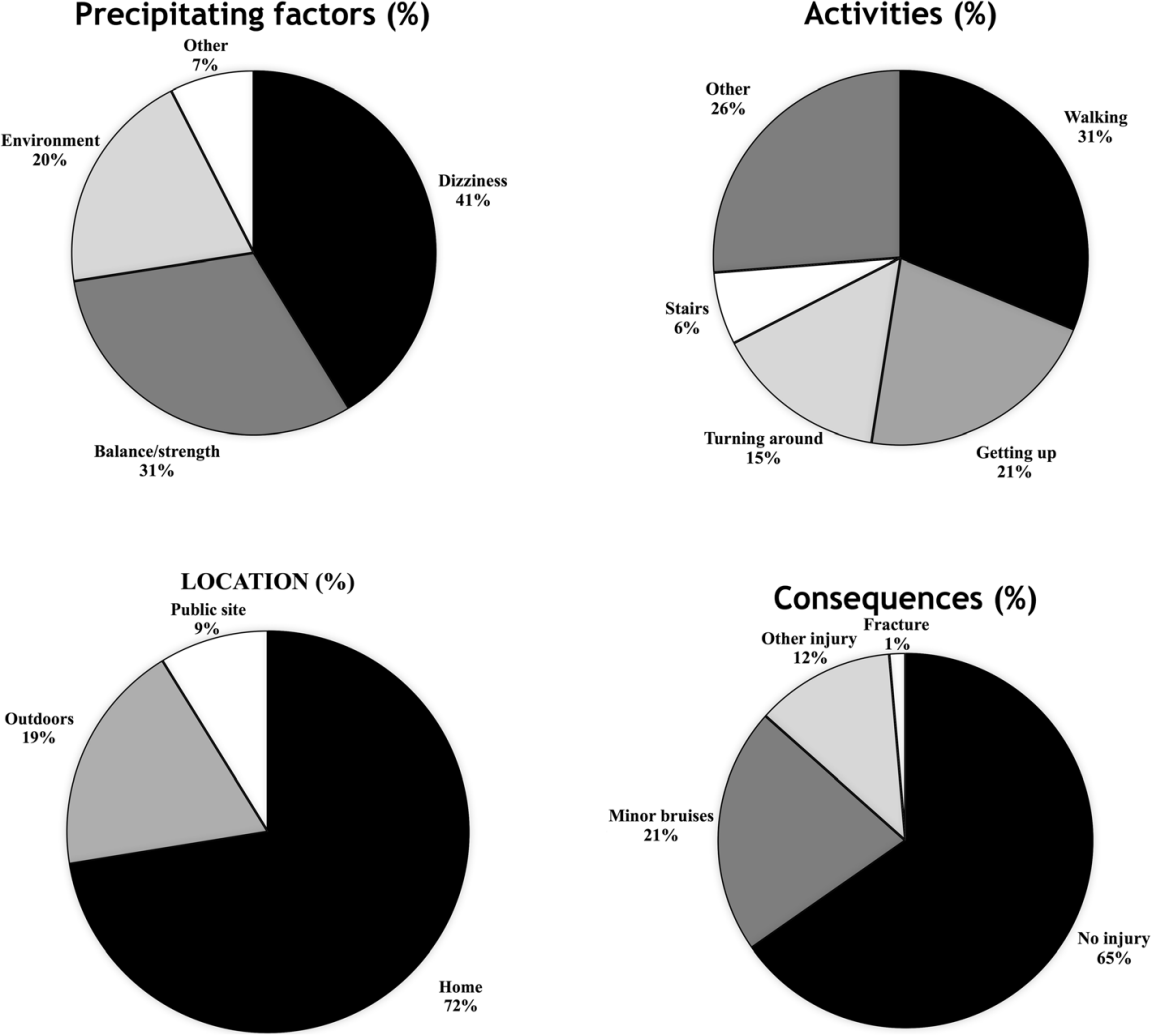
Characteristics of falls experienced by the study participants

A total number of 80 falls were recorded during the 12-month observational follow-up. Figure 1 shows the distribution of number of falls while Fig. 2 displays the characteristics of the falls experienced by study participants.

### Frailty and physical function

The frailty and physical function characteristics of study participants are summarised in Table 2. Fallers were more likely to meet the frailty component of self-reported exhaustion.

**Table 2:** Frailty and physical function characteristics of study participants: results are expressed as percentages for categorical variables and mean ± standard deviation for continuous data

Variables	All patients (69)	Fallers (26)	Non-fallers (43)	***P***-value
**Frailty components and phenotype**				
Low PA (%)	47.8	46.2	48.8	0.829
Low gait speed (%)	29	42.3	20.9	0.058
Low strength (%)	47.8	46.2	48.8	0.829
Exhaustion (%)	73.5	92.3	61.9	0.006
Unintentional weight loss (%)	17.6	28	11.6	0.108
Fried’s frailty phenotype (%)	37.7	50	30.2	0.101
**Objectively measured PA**				
Time spent standing (h)	2.4 ± 1.2	2.1 ± 1.1	2.5 ± 1.2	0.177
Daily steps (n°)	3086 ± 1810	2601 ± 1325	3366 ± 2004	0.144
Daily sit to stands (n°)	37.5 ± 12.6	38.3 ± 16.1	37.1 ± 10.3	0.894
**Strength**				
Handgrip (kg)	26.9 ± 9.5	24.5 ± 10	28.4 ± 9	0.101
Leg extension (kg)	20.1 ± 9	17.4 ± 8.3	21.8 ± 9.1	0.050
**Functional tests**				
Gait speed (m/s)	0.85 ± 0.26	0.78 ± 0.26	0.9 ± 0.25	0.067
TUG (s)	11.5 ± 4.8	13.3 ± 6.4	10.4 ± 3.4	0.055
CSTS-5 (s)	17.1 ± 8.9	19.6 ± 12.3	15.7 ± 6.3	0.355

Abbreviations: *PA* physical activity, *TUG* timed up and go test, *CSTS-5* 5 repetitions chair sit to stand test

### Cardiovascular function

The cardiovascular function characteristics of study participants at rest are summarised in Table 3, while the haemodynamic responses to HUT-60° are reported in Table 4.

**Table 3:** Cardiovascular function characteristics of study participants. Baroreflex and haemodynamic variables at rest: results are expressed as mean ± standard deviation

Variables	All patients (69)	Fallers (26)	Non-fallers (43)	P-value
**Baroreflex function**				
Up-ramps (n°)	20.4 ± 18.9	20.9 ± 22.9	20.1 ± 15.8	0.632
Down-ramps (n°)	18.4 ± 15.9	19.1 ± 18.3	17.9 ± 14.2	0.811
Total-ramps (n°)	38.8 ± 34.3	40 ± 40.7	38 ± 29.8	0.688
Up-events (n°)	4.5 ± 7.1	2.6 ± 3.7	5.7 ± 8.4	0.125
Down-events (n°)	4.8 ± 6.4	3.3 ± 4.5	5.7 ± 7.3	0.347
Total-events (n°)	9.3 ± 13	6 ± 7.9	11.4 ± 15.1	0.129
Up-BEI (%)	20.6 ± 25.2	14.6 ± 20.2	24.7 ± 27.7	0.165
Down-BEI (%)	27.9 ± 26.4	20.3 ± 22.7	32.9 ± 27.7	0.103
Total-BEI (%)	25.4 ± 25	17.7 ± 19.1	30.6 ± 27.4	0.078
BRS (ms/mmHg)	9.3 ± 7.1	10.3 ± 9.3	8.8 ± 5.9	0.806
**Haemodynamic variables**				
RRI (ms)	894.9 ± 163.1	867.7 ± 112.4	911.4 ± 186.8	0.240
HR (bpm)	69.5 ± 12	70.7 ± 9.3	68.8 ± 13.4	0.506
contSBP (mmHg)	124 ± 23.1	127 ± 26.2	122.1 ± 21.1	0.462
contDBP (mmHg)	77.8 ± 14.8	76.8 ± 12.5	78.4 ± 16.1	0.756
contmBP (mmHg)	97.2 ± 17.7	97.9 ± 16.9	96.8 ± 18.4	0.801
SV (ml)	64.4 ± 14.4	63.6 ± 12.1	64.8 ± 15.7	0.921
CO (L/min)	4.4 ± 1.2	4.5 ± 0.9	4.4 ± 1.3	0.416
TPR (dyne*s/cm^5^)	1777 ± 501	1716 ± 371	1816 ± 570	0.440
SI (ml/m^2^)	34.8 ± 8.8	35.2 ± 7.8	34.5 ± 9.5	0.455
CI (L/min*m^2^)	2.4 ± 0.7	2.5 ± 0.5	2.4 ± 0.8	0.167
TPRI(dyne*s*m^2^/cm^5^)	3330 ± 1037	3121 ± 710	3464 ± 1190	0.154
TFC (1/kΩ)	32.9 ± 10.8	34.6 ± 12.1	31.9 ± 10	0.365
OscSBP (mmHg)	128.2 ± 22	134.4 ± 26.3	124.8 ± 18.7	0.094
OscDBP (mmHg)	81 ± 13.9	82.4 ± 12.8	80.2 ± 14.5	0.553

Abbreviations: *Up-BEI* up-events baroreceptor effectiveness index, *Down-BEI* down-events baroreceptor effectiveness index, *Total-BEI* total-events baroreceptor effectiveness index, *BRS* baroreflex sensitivity, *RRI* R-R interval, *HR* heart rate, *contSBP* continuous systolic blood pressure, *contDBP* continuous diastolic blood pressure, *contmBP* continuous mean blood pressure, *SV* stroke volume, *CO* cardiac output, *TPR* total peripheral resistance, *SI* stroke index, *CI* cardiax index, *TPRI* total peripheral resistance index, *TFC* thoracic fluid content, *OscSBP* oscillometric systolic blood pressure, *OscDBP* oscillometric diastolic blood pressure

**Table 4:** Haemodynamic responses to HUT-60°. Difference between the variables averaged over 5 min of HUT-60° and the variables averaged over 5 min of supine recording: results are expressed as mean ± standard deviation

Variables	All patients (69)	Fallers (26)	Non-fallers (43)	***P***-value
RRI (ms)	−60.4 ± 66.8	−55.9 ± 71.1	−63 ± 65	0.514
HR (bpm)	6.2 ± 7.3	6.9 ± 9.6	5.8 ± 5.7	0.860
contSBP (mmHg)	3.9 ± 11.9	4.8 ± 14.9	3.4 ± 9.9	0.178
contDBP (mmHg)	6.3 ± 8.5	6.3 ± 10.6	6.3 ± 7.1	0.575
contmBP (mmHg)	5.3 ± 9.4	5.4 ± 12	5.2 ± 7.8	0.436
SV (ml)	−4.1 ± 13.8	−4.5 ± 12.7	−3.9 ± 14.5	0.693
CO (L/min)	0.01 ± 1	0 ± 0.9	0.02 ± 1	0.734
TPR (dyne*s/cm^5^)	98.3 ± 374.8	102.8 ± 367.4	95.5 ± 384.1	0.941
SI (ml/m^2^)	−2.3 ± 7.6	−2.3 ± 6.9	−2.3 ± 8	0.703
CI (L/min*m^2^)	−0.01 ± 0.5	0.01 ± 0.5	−0.01 ± 0.5	0.887
TPRI(dyne*s*m^2^/cm^5^)	173.7 ± 684	193.8 ± 655.3	161.3 ± 709.3	0.856
TFC (1/kΩ)	−1.9 ± 1.8	− 2 ± 1.9	−1.8 ± 1.8	0.632
OscSBP (mmHg)	−3.6 ± 9.8	−5.6 ± 10.9	−2.5 ± 9	0.249
OscDBP (mmHg)	−0.7 ± 6.9	−2.6 ± 7.5	0.3 ± 6.4	0.432

Abbreviations: *RRI* R-R interval, *HR*: heart rate, *contSBP* continuous systolic blood pressure, *contDBP* continuous diastolic blood pressure, *contmBP* continuous mean blood pressure, *SV* stroke volume, *CO* cardiac output, *TPR* total peripheral resistance, *SI* stroke index, *CI* cardiax index, *TPRI* total peripheral resistance index, *TFC* thoracic fluid content, *OscSBP* oscillometric systolic blood pressure, *OscDBP* oscillometric diastolic blood pressure

### Predictors of falls

The results from the negative binomial regression analyses are summarised in Table 5. All frailty/physical function and cardiovascular function factors reaching statistical significance in univariate analysis were adjusted in Model 1 for CRP and antidepressant use, as both of these clinical factors have been linked to an increased risk of falling in CKD-5 [1, 15]. Moreover, with concern for multi-collinearity (Additional file 1: Table S1), we only reported the baroreflex function index showing the highest correlation with number of falls (total-BEI). In fully adjusted analyses (Model 2), total-BEI, and the haemodynamic response of OscDBP to HUT-60° were associated with a higher number of falls.

**Table 5:** Negative binomial regression analysis: predictors of falls

Factors	Univariate		Multivariate			
			Model 1		Model 2	
	RR (95% CI)	***P***-value	RR (95% CI)	***P***-value	RR (95% CI)	***P***-value
**Frailty & physical function**						
Frailty (yes/no)	4.10 (1.60–10.51)	0.003	2.23 (0.85–5.87)	0.103	1.78 (0.70–4.51)	0.224
Daily steps (n°)	0.99 (0.99–1.00)	0.006	1.00 (0.99–1.00)	0.137	1.00 (0.99–1.00)	0.106
Daily sit to stands (n°)	0.96 (0.93–0.99)	0.042	0.98 (0.94–1.02)	0.387	0.99 (0.95–1.04)	0.707
Handgrip (kg)	0.94 (0.88–0.99)	0.034	0.97 (0.92–1.02)	0.216	0.98 (0.93–1.04)	0.448
Gait speed (m/s)	0.08 (0.01–0.62)	0.016	0.26 (0.03–2.08)	0.205	0.36 (0.04–3.32)	0.363
TUG (s)	1.16 (1.02–1.32)	0.021	1.08 (0.96–1.21)	0.220	1.03 (0.91–1.17)	0.643
**Cardiovascular function**						
Total-BEI (%)	0.96 (0.93–0.98)	<0.001	0.96 (0.94–0.99)	0.003	0.96 (0.94–0.99)	0.004
CO (L/min)	0.51 (0.27–0.98)	0.043	0.66 (0.37–1.15)	0.143	0.67 (0.39–1.13)	0.134
OscDBP (mmHg)	0.90 (0.83–0.98)	0.010	0.93 (0.87–0.99)	0.036	0.93 (0.87–0.99)	0.033

Abbreviations: *RR* rate ratio, *CI* confidence interval, *TUG* timed up and go test, *Total-BEI* total-events baroreceptor effectiveness index, *CO* cardiac output response to HUT-60°, *OscDBP* oscillometric diastolic blood pressure response to HUT-60°, *Model 1*: All factors are adjusted for CRP and antidepressant use, *Model 2* Frailty and physical function factors are adjusted for all variables in Model 1 and for Total-BEI. Cardiovascular function factors are adjusted for all variables in Model 1 and for frailty

Table 6 shows the goodness of fit (AIC) of the univariate regression models and its relative change following forced entry modelling of frailty and cardiovascular function (multivariate stage). The model composed of frailty and total-BEI resulted in a 19.1% decrease in AIC compared to frailty alone.

**Table 6:** Further analyses: goodness of fit of negative binomial regression models

Factors	Univariate			Multivariate^**a**^			
	RR (95% CI)	***P***-value	AIC	RR (95% CI)	***P***-value	AIC	ΔAIC
**Frailty and physical function**							
Frailty (yes/no)	4.10 (1.60–10.5)	0.003	185	2.53 (0.96–6.70)	0.060	149.6	***−19.1%***
Daily steps (n°)	0.99 (0.99–1.00)	0.006	142.3	1.00 (0.99–1.00)	0.036	113.2	***−20.4%***
Daily sit to stands (n°)	0.96 (0.93–0.99)	0.042	146.7	0.99 (0.95–1.03)	0.543	116.7	***− 20.4%***
Handgrip (kg)	0.94 (0.88–0.99)	0.034	188.6	0.97 (0.91–1.03)	0.330	151.8	***−19.5%***
Gait speed (m/s)	0.08 (0.01–0.62)	0.016	176.3	0.14 (0.02–1.39)	0.094	141.1	***−20.0%***
TUG (s)	1.16 (1.02–1.32)	0.021	171.7	1.10 (0.95–1.27)	0.226	138.5	***−19.3%***
**Cardiovascular function**							
Total-BEI (%)	0.96 (0.93–0.98)	<0.001	150.8	0.96 (0.94–0.99)	0.003	149.6	***−0.8%***
CO (L/min)	0.51 (0.27–0.98)	0.043	168.6	0.58 (0.33–1.04)	0.068	165.7	***−1.7%***
OscDBP (mmHg)	0.90 (0.83–0.98)	0.010	144.1	0.92 (0.85–0.99)	0.028	142.6	***−1.0%***

Abbreviations: *RR* rate ratio, *CI* confidence interval, *AIC* Akaike’s information criterion, *TUG* timed up and go test, *Total-BEI* total-events baroreceptor effectiveness index, *CO* cardiac output, *OscDBP* oscillometric diastolic blood pressure

^a^Frailty and physical function variables are adjusted by Total-BEI, cardiovascular function variables are adjusted by frailty

### Sensitivity analyses

**Fig. 3:**
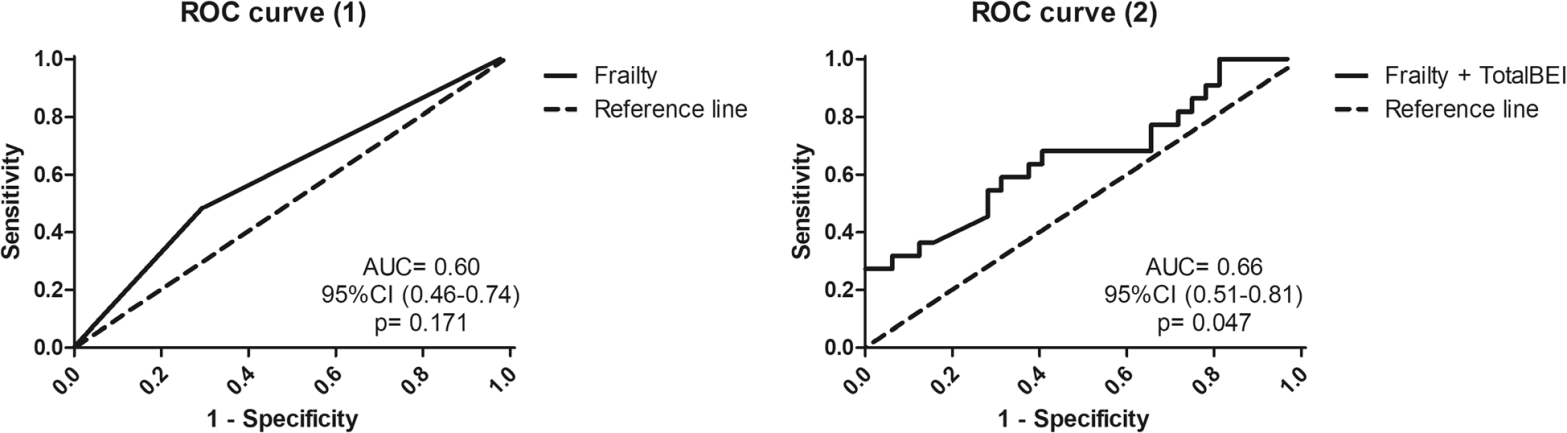
Sensitivity analyses: ROC curve analysis. Abbreviations: AUC: area under the curve; CI: confidence interval

Figure 3 shows the ROC curves of frailty alone and the model composed of frailty and total-BEI. Frailty alone did not discriminate significantly fallers from non-fallers, while adding the cardiovascular variable total-BEI resulted in a significant improvement of the AUC (+ 6%). In a further sensitivity analysis (Additional file 1: Table S2), we re-ran the negative binomial regression analysis adjusting Model 1 for diabetes, a well-established risk factor for falls in HD patients [1]. In this additional analysis, only total-BEI remained significantly associated with a higher number of falls (RR: 0.97, 95%CI: 0.95–0.99, *p* = 0.02).

## Discussion

We hypothesised that frailty and cardiovascular function would be associated with a higher number of falls in CKD-5 patients on HD. Additionally, we hypothesised that modelling the risk of falling by adding a cardiovascular function variable to frailty would improve the prediction of falls sustained over 12 months in this patient population.

The univariate negative binomial regression analysis revealed that frailty and other physical function measures, such as number of daily steps and sit to stands, handgrip strength, gait speed, and TUG were associated with a higher number of falls in the study population. In addition, cardiovascular function indices such as total-BEI, CO and OscDBP responses to HUT-60°, were also associated with a higher number of falls (Table 5). However, in multivariate analysis, only total-BEI and the OscDBP response to HUT-60° were significantly predictive of a greater number of falls. In addition, modelling the risk of falling by adding a cardiovascular function index (total-BEI) to frailty resulted in a 19.1% reduction in AIC, indicating a better goodness of fit, while adding frailty to cardiovascular indices such as total-BEI, CO and OscDBP responses to HUT-60°, hardly modified the AICs (− 0.8% to − 1.7%, Table 6). Moreover, the sensitivity analyses performed by means of ROC curve analysis revealed that adding total-BEI to the frailty model corresponded to a net 6% improvement of the AUC and resulted in a statistically significant prediction of falling status (Fig. 3).

The overall implications of these findings are that the addition of a baroreflex function index to an exclusively physical frailty-based model, did not only improve the prediction of falls, but it also highlighted the greater relative importance of a cardiovascular function index, implicated in the short-term regulation of BP, in predicting the occurrence of these falls. In particular, the forced entry modelling performed in Model 2 of the negative binomial regression analysis (Table 5), as well as the goodness of fit analysis (Table 6), suggest that lower baroreflex function and orthostatic decrements of BP may have an even higher impact than frailty on the prediction of falls in CKD-5 patients on HD.

The predominant role of these cardiovascular factors appeared to be also indirectly confirmed by the fall-related symptomatology reported by study participants (Fig. 2). The most common precipitating factors reported by fallers were dizziness/loss of consciousness (41.3% of falls), followed by loss of balance/unsteadiness of the legs (31.3% of falls), and environmental hazards (20% of falls). These symptoms seem to point out that the majority of falls were probably related to some underlying cardiovascular/haemodynamic type of mechanism. Because impairments of baroreflex function are linked to orthostatic decrements of BP [35], the dysregulation of BP during a sudden change in body position, or during prolonged standing may be one of the main mechanisms implicated in the aetiology of falls in CKD-5 patients on HD. Both of these factors predicted a higher number of falls in our study.

Another interesting finding was that BEI but not BRS was associated with a higher number of falls. The BEI is a measure that describes how often the baroreflex is activated [36], in contrast to the BRS which represents the baroreflex gain [37]. Therefore, we plausibly take the view that frequency, rather than intensity, of baroreflex activation may contribute more importantly to the increased risk of falling. Due to the fact that many spontaneous fluctuations of BP manifest suddenly throughout the day, as a result of changes in body position or more or less prolonged periods of standing [38], the baroreflex has to constantly adjust HR and peripheral resistance to maintain cardiac output. Thus, a reduced frequency of baroreflex activation (i.e. low BEI) would imply that sudden drops in BP are not coupled effectively with concomitant increases of HR, which may result in a suboptimal cerebral perfusion possibly leading to dizziness symptoms and, consequently, falls [39].

Many dialysis-specific or idiopathic risk factors may be involved in the dysregulation of BP which could possibly lead to dizziness symptoms and falls in this clinical population. First of all, the uremia-related cardiac autonomic dysfunction, characterised by alterations of the sympathetic, parasympathetic, and baroreflex components of the autonomic nervous system, is a factor that has been linked to symptomatic hypotension in CKD-5 patients [40]. Moreover, autonomic neuropathy is a common clinical complication of advanced diabetes, the most common cause of CKD [41], and it can also lead to a diminished response of BP to standing [42]. Secondly, dialysis patients are often treated with one or more antihypertensive drugs (83.8 and 51.5% of patients in our study). While this is often an unavoidable therapy for the treatment of hypertension, such antihypertensive polypharmacy has been linked to an increased risk of falling by inducing dizziness and postural hypotension [43].

Importantly, a systematic review with meta-analysis has recently concluded that exercise training can ameliorate autonomic regulation in CKD patients [44]. In particular, Petraki et al. [20] have shown that a seven-month intradialytic exercise programme led to improvements amounting to 27–35% in number of baroreceptor events and BEI in CKD-5 patients on HD. Since such measures were predictive of a higher number of falls in our study (Table 5), this raises the question as to whether an exercise training intervention may reduce the risk of falling by improving indices of autonomic function in people affected by CKD-5. Further research is warranted to explore this hypothesis as well as the cost-effectiveness of this potential intervention strategy.

The estimates of association between frailty and number of falls experienced emerging from this study are similar to those observed by previous research [4, 12]. In particular, McAdams-DeMarco et al. [4] explored the association between the Fried’s frailty phenotype and number of falls sustained over 6 months. The authors found that frailty was associated with a roughly 3.5 times higher number of falls in univariate Poisson regression analysis, an estimate that echoes the results from the univariate negative binomial regression analysis in our study (Table 5). Therefore, we conclude that our findings on the relationship between frailty and falls exhibit good external validity and may be generalised to the wider population of CKD-5 patients receiving HD.

Interestingly, the exploratory independent comparisons of fallers and non-fallers revealed that those who fell were more likely to use antidepressants (50% vs 23.8%) and had higher CRP levels (37.6 ± 59.2 mg/L vs 18.3 ± 32.7 mg/L). Because both antidepressant use and high CRP levels have been linked to a higher risk of falling by previous research conducted on HD patients [1, 15], we adjusted the analysis for these possible clinical confounders (Model 1). In this first model of multivariate analysis, neither frailty nor the additional physical function measures were significantly associated with a higher number of falls (Table 5). This finding further reinforces the observation that total-BEI and the OscDBP response to HUT-60° had a greater relative importance than frailty in predicting the number of falls experienced by study participants during the 12-month follow up.

It should be acknowledged that because the sample size was relatively small, we could not apply a more exhaustive multivariate negative binomial regression analysis to more robustly test the interrelationships between frailty/physical function, cardiovascular function, and falls. In addition, we should also acknowledge that, while orthostatic decrements of OscDBP were significantly associated with falls (Table 5), orthostatic decrements of OscSBP were not. However, in univariate negative binomial regression analysis, OscSBP decrements also exhibited a potential trend of association with falls (RR: 0.95, 95%CI: 0.89–1.01, *p* = 0.084), and it is possible that a larger sample size may have also resulted into better chances of detecting a significant association between OscSBP decrements and falls, by decreasing the chances of committing a type II error. The lack of relationship between contBP and falls may also be explained in light of the orthostatic challenge used in our study, as previous research has shown that the initial response of contBP to passive tilting is less pronounced or even absent compared to active stand procedures [45, 46]. Additionally, the relatively short duration of HUT-60° may be partially responsible for the apparent lack of difference in BP response during the orthostatic challenge in fallers compared with non-fallers (Table 4). Particularly, the five-minute HUT-60° protocol employed in this study was designed to investigate only the short-lived haemodynamic adjustments to orthostasis [18, 47]. Previous research has shown that, during a 15-min passive orthostatic challenge, elderly people with a history of falls tend to exhibit a larger drop of BP compared with non-fallers [39]. Therefore, it is plausible that a longer HUT-60° protocol may have revealed a larger decrement of both contBP and OscBP in fallers [46].

## Conclusions

This prospective cohort study indicates that by adding a cardiovascular index, implicated in the short-term regulation of BP, to a frailty-only model it was possible to improve significantly the prediction of falls in CKD-5 patients on HD. Baroreflex function, as assessed by means of BEI, and orthostatic decrements of BP showed a higher impact, and thus greater relative importance, than frailty in predicting number of falls in the study population. The clinical implications of these findings indicate that a simple non-invasive, time-efficient, assessment consisting of a continuous and simultaneous recording of BP and HR may be more useful than frailty assessment alone when predicting falls in HD patients. A high number of falls appear to be mediated by a degree of cardiovascular dysregulation, evidenced by the predominance of self-reported dizziness symptoms. We therefore recommend that interventions designed to improve baroreflex function/short-term regulation of BP should be considered when implementing falls prevention programmes in this patient population.

## Supplementary information

**Additional file 1: Table S1.** Multicollinearity analysis: correlations among baroreflex function variables; **Table S2.** Negative binomial regression analysis: sensitivity analyses.

## Data Availability

The datasets used and analysed during the current study are available from the corresponding author on reasonable request.

## Abbreviations

ACE: Angiotensin-converting enzyme
AIC: Akaike’s Information criterion
AngII: Angiotensin II
AUC: Area under the curve
BEI: Baroreceptor effectiveness index
BMI: Body mass index
BP: Blood pressure
BRS: Baroreflex sensitivity
Ca: Calcium
CCI: Charlson comorbidity index
CI: Cardiac index
CI: Confidence interval
CKD-5: Stage 5 chronic kidney disease
CO: Cardiac output
ContDBP: Continuous diastolic blood pressure
ContmBP: Continuous mean blood pressure
ContSBP: Continuous systolic blood pressure
CRP: C-reactive protein
CSTS-5: 5 repetitions chair sit to stand test
CVD: Cardiovascular disease
Down-BEI: Down-events baroreceptor effectiveness index
ECG: Electrocardiography
Hb: Hemoglobin
HD: Haemodialysis
HR: Heart rate
HUT-60°: Head-up tilt test at 60°
ICG: Impedance cardiography
IPAQ-SF: Short-form international physical activity
K: Potassium
K-S: Kolmogorov-Smirnov test
Na: Sodium
NHS: National Health Service
OscDBP: Oscillometric diastolic blood pressure
OscSBP: Oscillometric systolic blood pressure
PA: Physical activity
PTH: Parathyroid hormone
ROC: Receiver operating characteristic
RR: Rate ratio
RRI: R-R interval
SF-36: Short form health survey
SI: Stroke index
SV: Stroke volume
TFC: Thoracic fluid content
Total-BEI: Total-events baroreceptor effectiveness index
TPR: Total peripheral resistance
TPRI: Total peripheral resistance index
TUG: 3-m timed up and go test
Up-BEI: Up-events baroreceptor effectiveness index
URR: Urea reduction ratio

## References

[1] Desmet C, Beguin C, Swine C, Jadoul M, Université Catholique de Louvain collaborative group (2005). Falls in hemodialysis patients: prospective study of incidence, risk factors, and complications. Am J Kidney Dis.

[2] Cook WL, Tomlinson G, Donaldson M, Markowitz SN, Naglie G, Sobolev B (2006). Falls and fall-related injuries in older dialysis patients. Clin J Am Soc Nephrol.

[3] López-Soto PJ, De Giorgi A, Senno E, Tiseo R, Ferraresi A, Canella C (2015). Renal disease and accidental falls: a review of published evidence. BMC Nephrol.

[4] McAdams-DeMarco MA, Suresh S, Law A, Salter ML, Gimenez LF, Jaar BG (2013). Frailty and falls among adult patients undergoing chronic hemodialysis: a prospective cohort study. BMC Nephrol.

[5] Roberts R, Jeffrey C, Carlisle G, Brierley E (2007). Prospective investigation of the incidence of falls, dizziness and syncope in haemodialysis patients. Int Urol Nephrol.

[6] O'Loughlin JL, Robitaille Y, Boivin JF, Suissa S (1993). Incidence of and risk factors for falls and injurious falls among the community-dwelling elderly. Am J Epidemiol.

[7] Abdel-Rahman EM, Yan G, Turgut F, Balogun RA (2011). Long-term morbidity and mortality related to falls in hemodialysis patients: role of age and gender – a pilot study. Nephron Clin Pract.

[8] Li M, Tomlinson G, Naglie G, Cook WL, Jassal SV (2008). Geriatric comorbidities, such as falls, confer an independent mortality risk to elderly dialysis patients. Nephrol Dial Transplant.

[9] Kojima G (2017). Prevalence of frailty in end-stage renal disease: a systematic review and meta-analysis. Int Urol Nephrol.

[10] Fried LP, Tangen CM, Walston J, Newman AB, Hirsch C, Gottdiener J (2001). Cardiovascular health study collaborative research group. Frailty in older adults: evidence for a phenotype. J Gerontol A Biol Sci Med Sci.

[11] Koufaki P, Greenwood S, Painter P, Mercer T (2015). The BASES expert statement on exercise therapy for people with chronic kidney disease. J Sports Sci.

[12] Kutner NG, Zhang R, Huang Y, Wasse H (2014). Falls among hemodialysis patients: potential opportunities for prevention?. Clin Kidney J.

[13] Delgado C, Shieh S, Grimes B, Chertow GM, Dalrymple LS, Kaysen GA (2015). Association of Self-Reported Frailty with falls and fractures among patients new to dialysis. Am J Nephrol.

[14] Wang AY, Sherrington C, Toyama T, Gallagher MP, Cass A, Hirakawa Y (2017). Muscle strength, mobility, quality of life and falls in patients on maintenance haemodialysis: a prospective study. Nephrology (Carlton).

[15] Kono K, Nishida Y, Yabe H, Moriyama Y, Mori T, Shiraki R (2018). Development and validation of a fall risk assessment index for dialysis patients. Clin Exp Nephrol.

[16] Foley RN, Parfrey PS (1997). Cardiac disease in chronic uremia: clinical outcome and risk factors. Adv Ren Replace Ther.

[17] Roberts RG, Kenny RA, Brierley EJ (2003). Are elderly haemodialysis patients at risk of falls and postural hypotension?. Int Urol Nephrol.

[18] Zanotto T, Mercer TH, van der Linden ML, Traynor JP, Petrie CJ, Doyle A (2018). Baroreflex function, haemodynamic responses to an orthostatic challenge, and falls in haemodialysis patients. PLoS One.

[19] Polinder-Bos HA, Emmelot-Vonk MH, Gansevoort RT, Diepenbroek A, Gaillard CA (2014). High fall incidence and fracture rate in elderly dialysis patients. Neth J Med.

[20] Petraki M, Kouidi E, Grekas D, Deligiannis A (2008). Effects of exercise training during hemodialysis on cardiac baroreflex sensitivity. Clin Nephrol.

[21] Lamb SE, Jørstad-Stein EC, Hauer K, Becker C, prevention of falls network Europe and outcomes consensus group (2005). Development of a common outcome data set for fall injury prevention trials: the prevention of falls network Europe consensus. J Am Geriatr Soc.

[22] Craig CL, Marshall AL, Sjöström M, Bauman AE, Booth ML, Ainsworth BE (2003). International physical activity questionnaire: 12-country reliability and validity. Med Sci Sports Exerc.

[23] Johansen KL, Chertow GM, Jin C, Kutner NG (2007). Significance of frailty among dialysis patients. J Am Soc Nephrol.

[24] Shumway-Cook A, Brauer S, Woollacott M (2000). Predicting the probability for falls in community-dwelling older adults using the timed up & go test. Phys Ther.

[25] Guralnik JM, Simonsick EM, Ferrucci L, Glynn RJ, Berkman LF, Blazer DG (1994). A short physical performance battery assessing lower extremity function: association with self-reported disability and prediction of mortality and nursing home admission. J Gerontol.

[26] Aagaard P, Simonsen EB, Andersen JL, Magnusson SP, Bojsen-Møller F, Dyhre-Poulsen P (2000). Antagonist muscle coactivation during isokinetic knee extension. Scand J Med Sci Sports.

[27] Koufaki P, Kouidi E (2010). Current best evidence recommendations on measurement and interpretation of physical function in patients with chronic kidney disease. Sports Med.

[28] Greenwood SA, Koufaki P, Mercer TH, Rush R, O'Connor E, Tuffnell R (2015). Aerobic or resistance training and pulse wave velocity in kidney transplant recipients: a 12-week pilot randomized controlled trial (the exercise in renal transplant [ExeRT] trial). Am J Kidney Dis.

[29] Lyden K, Keadle SK, Staudenmayer J, Freedson PS (2017). The activPALTM accurately classifies activity intensity categories in healthy adults. Med Sci Sports Exerc.

[30] Gratze G, Fortin J, Holler A, Grasenick K, Pfurtscheller G, Wach P (1998). A software package for non-invasive, real-time beat-to-beat monitoring of stroke volume, blood pressure, total peripheral resistance and for assessment of autonomic function. Comput Biol Med.

[31] Jeleazcov C, Krajinovic L, Münster T, Birkholz T, Fried R, Schüttler J (2010). Precision and accuracy of a new device (CNAP™) for continuous non-invasive arterial pressure monitoring: assessment during general anaesthesia. Br J Anaesth.

[32] Ilies C, Bauer M, Berg P, Rosenberg J, Hedderich J, Bein B (2012). Investigation of the agreement of a continuous non-invasive arterial pressure device in comparison with invasive radial artery measurement. Br J Anaesth.

[33] Pinna GD, La Rovere MT, Maestri R, Mortara A, Bigger JT, Schwartz PJ (2000). Comparison between invasive and non-invasive measurements of baroreflex sensitivity; implications for studies on risk stratification after a myocardial infarction. Eur Heart J.

[34] Pitzalis M, Parati G, Massari F, Guida P, Di Rienzo M, Rizzon B (2003). Enhanced reflex response to baroreceptor deactivation in subjects with tilt-induced syncope. J Am Coll Cardiol.

[35] Mattace-Raso FU, van den Meiracker AH, Bos WJ, van der Cammen TJ, Westerhof BE, Elias-Smale S (2007). Arterial stiffness, cardiovagal baroreflex sensitivity and postural blood pressure changes in older adults: the Rotterdam study. J Hypertens.

[36] Di Rienzo M, Parati G, Castiglioni P, Tordi R, Mancia G, Pedotti A (2001). Baroreflex effectiveness index: an additional measure of baroreflex control of heart rate in daily life. Am J Physiol Regul Integr Comp Physiol.

[37] Pinna GD, Maestri R, La Rovere MT (2015). Assessment of baroreflex sensitivity from spontaneous oscillations of blood pressure and heart rate: proven clinical value?. Physiol Meas.

[38] Silvani A, Calandra-Buonaura G, Johnson BD, van Helmond N, Barletta G, Cecere AG (2017). Physiological mechanisms mediating the coupling between heart period and arterial pressure in response to postural changes in humans. Front Physiol.

[39] Shaw BH, Loughin TM, Robinovitch SN, Claydon VE (2015). Cardiovascular responses to orthostasis and their association with falls in older adults. BMC Geriatr.

[40] Robinson TG, Carr SJ (2002). Cardiovascular autonomic dysfunction in uremia. Kidney Int.

[41] GBD 2015 Mortality and Causes of Death Collaborators (2016). Global, regional, and national life expectancy, all-cause mortality, and cause-specific mortality for 249 causes of death, 1980–2015: a systematic analysis for the Global Burden of Disease Study 2015. Lancet.

[42] Vinik AI, Casellini C, Parson HK, Colberg SR, Nevoret ML (2018). Cardiac autonomic neuropathy in diabetes: a predictor of Cardiometabolic events. Front Neurosci.

[43] Tinetti ME, Han L, Lee DS, McAvay GJ, Peduzzi P, Gross CP (2014). Antihypertensive medications and serious fall injuries in a nationally representative sample of older adults. JAMA Intern Med.

[44] Heiwe S, Jacobson SH (2014). Exercise training in adults with CKD: a systematic review and meta-analysis. Am J Kidney Dis.

[45] Imholz BP, Settels JJ, van der Meiracker AH, Wesseling KH, Wieling W (1990). Non-invasive continuous finger blood pressure measurement during orthostatic stress compared to intra-arterial pressure. Cardiovasc Res.

[46] van Wijnen VK, Finucane C, Harms MPM, Nolan H, Freeman RL, Westerhof BE (2017). Noninvasive beat-to-beat finger arterial pressure monitoring during orthostasis: a comprehensive review of normal and abnormal responses at different ages. J Intern Med.

[47] Brignole M, Moya A, de Lange FJ, Deharo JC, Elliott PM, Fanciulli A (2018). 2018 ESC guidelines for the diagnosis and management of syncope. Eur Heart J.

