# Supplementary material for: The relative importance of frailty, physical and cardiovascular function as exercise-modifiable predictors of falls in haemodialysis patients: a prospective cohort study
Source: BMC Nephrol. 2020 Mar 14;21:99. doi: 10.1186/s12882-020-01759-z (PMC7071740; doi:10.1186/s12882-020-01759-z)
Supplement: Supplementary file 1 — Additional file 1: Table S1. Multicollinearity analysis: correlations among baroreflex function variables; Table S2. Negative binomial regression analysis: sensitivity analyses. [file 12882_2020_1759_MOESM1_ESM.pdf]

Table S1. Multicollinearity analysis: correlations among baroreflex function variables.

**Variables**	*1*	*2*	*3*	*4*	*5*	*6*	*7*
1. Up-events (n°)	1.000	.808**	.931**	.948**	.620**	.775**	-.265*
2. Down-events (n°)	.808**	1.000	.948**	.773**	.830**	.812**	-.185
3. Total-events (n°)	.931**	.948**	1.000	.875**	.775**	.838**	-.268*
4. Up-BEI (%)	.948**	.773**	.875**	1.000	.642**	.816**	-.256
5. Down-BEI (%)	.620**	.830**	.775**	.642**	1.000	.931**	-.297*
6. Total-BEI (%)	.775**	.812**	.838**	.816**	.931**	1.000	-.324*
7. Falls (n°)	-.265*	-.185	-.268*	-.256	-.297*	-.324*	1.000

Abbreviations: Up-BEI: up-events baroreceptor effectiveness index; Down-BEI: down-events baroreceptor effectiveness index; Total-BEI: total-events baroreceptor effectiveness index; * indicates a significant correlation (p-value < .05); ** indicates a significant correlation (p-value < .01).

Table S2. Negative binomial regression analysis: sensitivity analyses.

	**Univariate**		**Multivariate**			
			**Model 1**		**Model 2**	
**Factors**	**RR (95% CI)**	**P-value**	**RR (95% CI)**	**P-value**	**RR (95% CI)**	**P-value**
**Frailty & physical function**						
Frailty (yes/no)	4.10 (1.60-10.51)	0.003	1.87 (0.74-4.76)	0.187	1.58 (0.63-3.99)	0.330
Daily steps (n°)	0.99 (0.99-1.00)	0.006	1.00 (0.99-1.00)	0.575	1.00 (0.99-1.00)	0.686
Daily sit to stands (n°)	0.96 (0.93-0.99)	0.042	1.01 (0.97-1.06)	0.647	1.03 (0.97-1.08)	0.328
Handgrip (Kg)	0.94 (0.88-0.99)	0.034	0.96 (0.91-1.01)	0.096	0.96 (0.91-1.01)	0.142
Gait speed (m/s)	0.08 (0.01-0.62)	0.016	0.39 (0.05-2.95)	0.361	0.50 (0.05-4.59)	0.537
TUG (s)	1.16 (1.02-1.32)	0.021	1.06 (0.95-1.19)	0.299	0.02 (0.90-1.15)	0.817
**Cardiovascular function**						
Total-BEI (%)	0.96 (0.93-0.98)	<0.001	0.97 (0.95-0.99)	0.020	0.97 (0.95-0.99)	0.020
CO (L/min)	0.51 (0.27-0.98)	0.043	0.65 (0.40-1.06)	0.084	0.68 (0.43-1.07)	0.098
OscDBP (mmHg)	0.90 (0.83-0.98)	0.010	0.96 (0.88-1.04)	0.344	0.99 (0.91-1.07)	0.736

Abbreviations: RR: rate ratio; CI: confidence interval; TUG: timed up and go test; Total-BEI: total-events baroreceptor effectiveness index; CO: cardiac output response to HUT-60°; OscDBP:

oscillometric diastolic blood pressure response to HUT-60°; Model 1: All factors are adjusted for diabetic status and antidepressant use; Model 2: Frailty and physical function factors are adjusted for all variables in Model 1 and for Total-BEI. Cardiovascular function factors are adjusted for all variables in Model 1 and for frailty.
